# PAR1 and PAR4 exert opposite effects on tumor growth and metastasis of esophageal squamous cell carcinoma via STAT3 and NF-κB signaling pathways

**DOI:** 10.1186/s12935-021-02354-4

**Published:** 2021-11-29

**Authors:** Jia Zhao, Guangyu Jin, Xudong Liu, Kai Wu, Yang Yang, Zhanfeng He, Donglei Liu, Chunyang Zhang, Dengyan Zhu, Jia Jiao, Xiangnan Li, Song Zhao

**Affiliations:** Department of Thoracic Surgery, The First Affiliated Hospital of Zhengzhou University, Zhengzhou, Henan China

**Keywords:** Esophageal squamous cell carcinoma, Protease-activated receptors, Cancer metastasis, STAT3/NF-κB

## Abstract

**Background:**

Esophageal carcinogenesis is a multifactorial process in which genetic and environmental factors interact to activate intracellular signals, leading to the uncontrolled survival and growth of esophageal squamous cell carcinoma (ESCC) cells. The intracellular pathways of ESCC cells could be regulated by proteinase activated-receptors (PARs), which are comprised of four receptors (i.e., PAR-1, PAR-2, PAR-3, and PAR-4). Therefore, the function and possible mechanism of PAR1 and PAR4 in the progression of ECSS were explored in our study.

**Methods:**

First, we detected the expression levels of PAR1 and PAR4 in 27 cases of ESCC specimens and cell lines by RT-qPCR, IHC and western blot. Meanwhile, the correlation between PAR1/PAR4 expression levels, clinicopathological characteristics, and disease free survival was analyzed. Then, we constructed PAR1/PAR4 knockdown cell models and investigated the role of PAR1/PAR4 knockdown on the proliferation, apoptosis, changes of calcium flow, and metastasis of ESCC cells via MTT, flow cytometry, transwell and wound healing assays in vitro. Further, an experimental metastasis model in vivo was established to explore the role of stable PAR1/PAR4 knockdown on the growth and metastasis of ESCC cells. Finally, the role of nSMase2 in the activation of NF-κB induced by PAR4 and the role of NF-κB and STAT3 signaling pathways in the PAR1/PAR4-mediated tumor promoting or suppressive functions were measured by immunoprecipitation, western blot and immunofluorescence assays.

**Results:**

First, the integrated results demonstrated the expression levels of PAR1 and PAR4 are inversely proportional in ESCC. PAR1 potently enhanced tumor growth and metastasis, while PAR4 had an inhibitory effect. Further, the co-activation of STAT3 and NF-κB was involved in the PAR1 activation-induced tumor promoting effect, while only NF-κB participated in the PAR4 activation-induced tumor inhibitory effect in ESCC. To be specific, FAK/PI3K/AKT/STAT3/NF-κB signaling mediated PAR1 activation-induced tumor promoting effect and nSMase2/MAPK/NF-κB signaling mediated PAR4 activation-induced tumor inhibitory effect.

**Conclusions:**

Overall, the study has provided new insights into the potential implication of PAR1 and PAR4 in the pathogenesis of ESCC. Besides, FAK/PI3K/AKT/STAT3/NF-κB and nSMase2/MAPK/NF-κB pathways may be novel targets for regulating tumor growth and metastasis in ESCC patients.

**Supplementary Information:**

The online version contains supplementary material available at 10.1186/s12935-021-02354-4.

## Background

Esophageal cancer (EC) is one of the most aggressive gastrointestinal cancer [1]. East Asian countries, including China, are the areas of high incidence of esophageal squamous cell carcinoma (ESCC) [2]. Due to the lack of early clinical symptoms, ESCC is often diagnosed during its advanced stages and becomes a highly aggressive malignancy with poor prognosis [3]. Therefore, it is crucial to confirm a potentially valuable and early diagnostic indicator.

Many studies have demonstrated the hemostatic components may participate in the progress of cancer development [4–6]. Coagulant factors, like tissue factor (TF) and thrombin, may be generated in the tumor microenvironment and can induce cell signaling by activating protease-activated receptors (PARs) [7, 8]. PARs are a unique family of G-protein coupled receptors, which play important roles in vascular physiology, neural tube closure, hemostasis, and inflammation [9]. Some of their members are considered to promote cancer metastasis [10, 11]. There are four members of PARs in humans, that is, PAR1, PAR2, PAR3 and PAR4 [12]. Currently, studies have shown that PAR1 is positively expressed in 68.2% of tumor tissues derived from EC patients but not in normal esophageal squamous epithelium. PAR1 overexpression is significantly related to tumor metastasis (TNM) stage and regional lymph node involvement [13]. High expression of PAR2 in ESCC are also reported [14]. On the contrary, PAR4 is frequently down-regulated in ESCC specimens which is partly due to the hypermethylation of the PAR4 promoter [15].

PAR1 and PAR4 are both thrombin receptors and share the same ligands and the mechanism of activation, however, their expression levels are inversely proportional in ESCC cells. This discovery aroused our interest in exploring the role of PAR1 and PAR4 in the development of ECSS, which has not been clearly explored yet.

In this study, we found that PAR1 induced the development of ESCC through the co-activation pathway of STAT3 and NF-κB, and the inhibition of the AKT pathway also played an important role. On the contrary, PAR4 had an inhibitory effect on the development of ESCC by activating ERK and NF-κB rather than STAT3. Overall, this study provides a new insight into the potential implication of PAR1 and PAR4 in the pathogenesis of ESCC.

## Methods

### Cells and reagents

Human esophageal epithelial cells (HEEC) were purchased from ScienCell. EC109, Kyse150, Kyse140 and TE-1 cell lines were purchased from American Type Culture Collection (ATCC, Rockville, MD, USA) and cultured in RPMI-1640 (Cat. no. 72400120, Gibco, CA, USA) containing 10% fetal bovine serum (FBS) and 1% penicillin-streptomycin (P/S). Cells were maintained in 37 °C humidified incubator containing 5% (v/v) CO_2_.

### Clinical samples

We have clinically collected 27 cases of ESCC specimens and paired normal adjacent tissues from ESCC patient not treated with chemotherapy. The normal adjacent tissues are 2–2.5 cm away from the lesion. The investigation was approved by the Ethics Committee of The First Affiliated Hospital of Zhengzhou University and written consents were provided by all patients. The clinical diagnosis was confirmed by two experienced pathologists without discrepancy.

### Animals

C57BL/6J mice were purchased from JSJ laboratories (Shanghai, China). All mice were housed and bred in The First Affiliated Hospital of Zhengzhou University Research Vivarium. Animal experiments were conducted using protocols approved by IACUC of The First Affiliated Hospital of Zhengzhou University. 6- to 8-week-old mice were housed in a clean grade room with 21 ± 1 ℃ temperature and 60 ± 5% humidity. Animals were monitored daily according to humane endpoint guidelines by animal husbandry staff and in-house veterinarians. Mice were euthanized by intraperitoneal injection of 150 mg/kg sodium pentobarbital 50 days after subcutaneous injection with tumor cells, and then the heartbeat of the mice was examined before anatomy.

### The construction of knockdown and overexpression plasmids

PAR1 or PAR4 short hairpin RNA (shRNA, synthesized by Gene Pharma, CN) were utilized to knock down the protein levels of PAR1 or PAR4 in ESCC cells. Sequences of shRNA targeting PAR1, PAR4 and Akt were designed and cloned into pSicoR-GFP vector (Addgene, Cambridge, MA). For overexpression plasmids, gRNA for PAR1 or PAR4 was designed at the website (http://sam.genome-engineering.org/database/). gRNA oligo was inserted into lenti gRNA (MS2)-puro plasmid followed by Oligo annealing, digestion with BsmBI, ligation, transformation and plasmid extraction. 72 h after transfection, the lentivirus was collected and filtered with 0.45 μm filters. After infection with the lentivirus for 24 h, culture medium was refreshed for further treatment and detection.

### In vivo spontaneous metastasis model

To investigate the role of PAR1/PAR4 in ESCC metastasis, 6 to 8-week-old C57BL/6J mice were randomly divided into six groups (n = 6 each group). Mice were subcutaneously injected with 1 × 10^5^ sham-transfected TE-1 cells (NC), PAR1 or PAR4 knockdown TE-1 cells (PAR1 KD/PAR4 KD), and PAR1 or PAR4 overexpression TE-1 cells (PAR1 OE/PAR4 OE), respectively. After 50 days, the metastatic nodules on the lung surface were counted and the primary tumor volume was calculated. To investigate the role of STAT3 in PAR1-activating tumor metastasis, 6 to 8-week-old BALB/c mice were randomly divided into three groups (n = 6 each group). Mice were subcutaneously injected with 1 × 10^5^ sham-transfected Kyse140 cells. PAR1 AP and STAT3 inhibitor S3I-201 were transfused into mice via tail vein. After 50 days, the metastatic nodules on the lung surface were counted and the primary tumor volume was calculated.

### Immunofluorescence

ESCC cells were blocked with 3% BSA (Sigma) for 1 h at room temperature (RT), then incubated overnight with anti-p-STAT3 antibody (Abcam, ab5694), anti-STAT3 antibody (Abcam, ab5694), and anti-p-NF-κB antibody (Abcam, ab5694). After washing with PBS, cells were incubated with Cy3-labeled anti rabbit IgG or FITC-labeled anti rabbit IgG for 1 h at RT. The fluorescence images were captured using confocal microscope (Nikon).

### Western blot

Cells were lysed with cold RIPA buffer plus phosphatase and protein inhibitors, then centrifuged at 12,000 rpm for 30 min to remove cell debris. The total protein concentrations were determined by the Bradford method. 40 µg of total protein were separated on SDS-PAGE and electro-transferred to a polyvinylidene difluoride (PVDF) membrane. Membranes were blocked with 5% bovine serum albumin (BSA) for 1 h and then incubated overnight at 4 °C with different primary antibodies. After being incubated with HRP-conjugated second antibody for 1 h at RT, the blots were detected using Tanon Imaging System (5200 S).

### Flow cytometry detection of cell apoptosis

Apoptosis assay was performed following the instruction of Annexin V-FITC Apoptosis Detection Kit (C1062M, Beyotime, China) and detected by a Becton-Dickinson FACS Canto II instrument.

### MTT assay

ESCC cells were incubated with 5 mg/ml 3-(4,5‐dimethylthiazol‐2‐yl)‐2,5‐diphenyltetrazolium bromide (MTT; Sigma‐Aldrich) at 37 °C for 4 h; 100 µl dimethyl sulfoxide was then added. The optical density (OD) was measured at both 492 nm and 630 nm wavelength; Cell viability was calculated according to the following formula: cell viability = [OD value of treatment group-OD value of background]/ [OD value of control group-OD value of background] × 100%.

### Real-time RT-PCR

Total RNA was isolated from cells with TRIzol reagent (Invitrogen, USA). cDNA was synthesized with the PrimeScript RT Reagent Kit (TransGen Biotech, China). qRT-PCR analysis was performed following the instruction of SYBR Green Premix Ex Taq (Takara, China). mRNA levels were normalized with GAPDH. The sequences of forward (F) and reverse (R) primers were as follows: GAPDH-F, 5′-GACCTGACCTGCCGTCTA-3′; GAPDH-R, 5′-GGAGTGGGTGTCGCTGT-3′; PAR1-F, 5′-ATGGACATTTTTATAGTTAAGGA-3′; PAR1-R, 5′-AAGGTATCTGATTTGATATAA-3′; PAR4-F, 5′-CGATCTGCTCGTCCGCCTCGG-3′; PAR4-R, 5′-TAGCACCCCCAACTCTGCTCC-3′.

### Transwell and wound healing assays

For Transwell assay, cultured ESCC cells were added to upper chambers (10^4^ cells per well) of 8 μm-24 well Transwell culture dishes with 10% FBS culture medium in lower chambers. After 24 h, cells were rinsed with calcium free PBS and fixed with methanol, then stained with crystal violet. Tumor cells passed through the membranes were evaluated in five randomly selected fields, then dissolved in methanol and quantified by microplate reader OD 405 nm. The wound healing was carried out as previously described [16].

### Bioinformatics analysis

The data used for Disease Free Survival (DFS) weredownloaded from The Cancer Genome Atlas (TCGA, https://tcga-data.nci.nih. gov/docs/publications/tcga/). The Kaplan–Meier method with the log-rank test was applied to calculate the DFS rate for comparison between different groups. A corrected *P*-value < 0.05 was adopted as the standard for judging the enrichment of cluster genes with statistical significance.

### Statistical analysis

Statistical analysis was performed using Graphpad Prism 6.0 (GraphPad Software Inc., CA, USA). All experiments were carried out at least three times, and the results were presented as mean ± standard deviation (SD). Statistical significance between two groups was assessed using the one-way analysis of variance (ANOVA) followed by Dunnett’s post hoc test. Survival curves were plotted using the Kaplan–Meier method and compared using the log-rank test. *P*-values < 0.05 were considered statistically significant.

## Results

### The expression levels of PAR1 and PAR4 are inversely proportional in ESCC

**Fig. 1:**
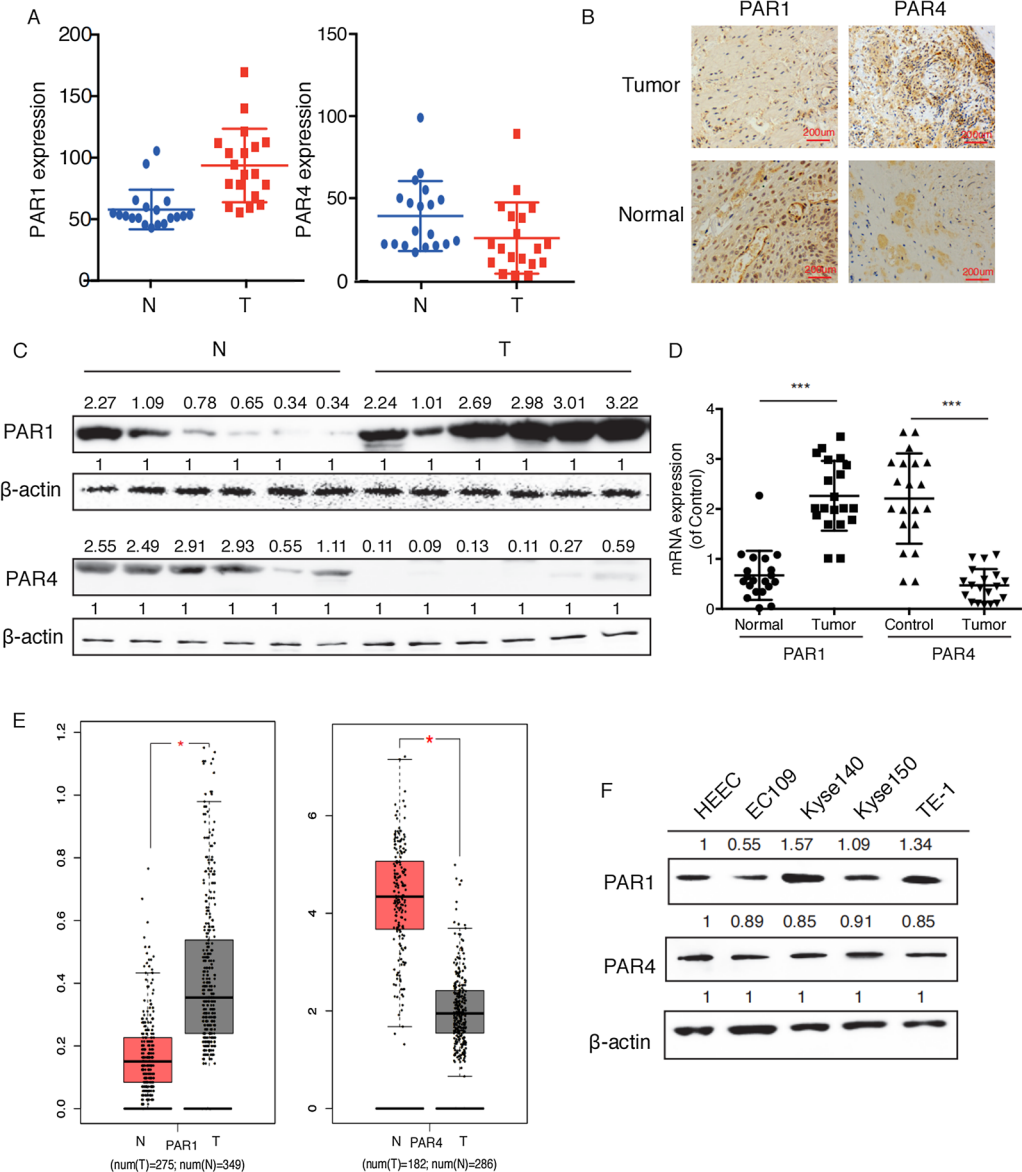
The expression levels of PAR1 and PAR4 are inversely proportional in ESCC. **A **IHC detected the expression of PAR1 and PAR4 in ESCC tissues and paired para-carcinoma tissues and representative photomicrographs of immunohistochemical staining were shown in **B**. **C** Western blot and **D** RT-PCR detected the expression of PAR1 and PAR4 at protein level and mRNA level, respectively. **E** PAR1 and PAR4 mRNA expression data in ESCC patients were collected from TCGA database. **F** Western blot detected the expression of PAR1 and PAR4 in HEEC and ESCC cell lines (EC109, Kyse140, Kyse150 and TE-1). Data are presented as mean ± SD from three independent experiments; comparison between two groups, *P < 0.05; **P < 0.01; ***P < 0.001

We clinically collected 27 cases of ESCC tissues and paired normal adjacent tissues. The results of qPCR and IHC showed that the expression of PAR1 in tumors was higher, while the expression of PAR4 was lower, than that in paired normal adjacent tissues (Fig. 1A, B). The similar results were also found with western blot and RT-PCR (Fig. 1C, D). Next, we analyzed the expression of PAR1 and PAR4 in ESCC tissues and normal adjacent tissues according to the data collected from TCGA database. The bioinformatic results from GEPIA (http://gepia.cancer-pku.cn/) indicated that the levels of PAR4 are lower, while the levels of PAR1 are significantly higher in tumor tissues than in normal tissues (Fig. 1E). Then, the expression of PAR1 and PAR4 was detected in human normal esophageal epithelial cells (HEEC) and ESCC cell lines EC109, Kyse140, Kyse150 and TE-1. Western blot results suggested that compared with HEEC, PAR4 was significantly downregulated in all four cancer cell lines, while PAR1 was overexpressed in two of the cancer cell lines (Kyse140 and TE-1) (Fig. 1F).

#### PAR1 promoted the growth and metastasis of ESCC cell lines, while PAR4 had an inhibitory effect in vitro

**Fig. 2:**
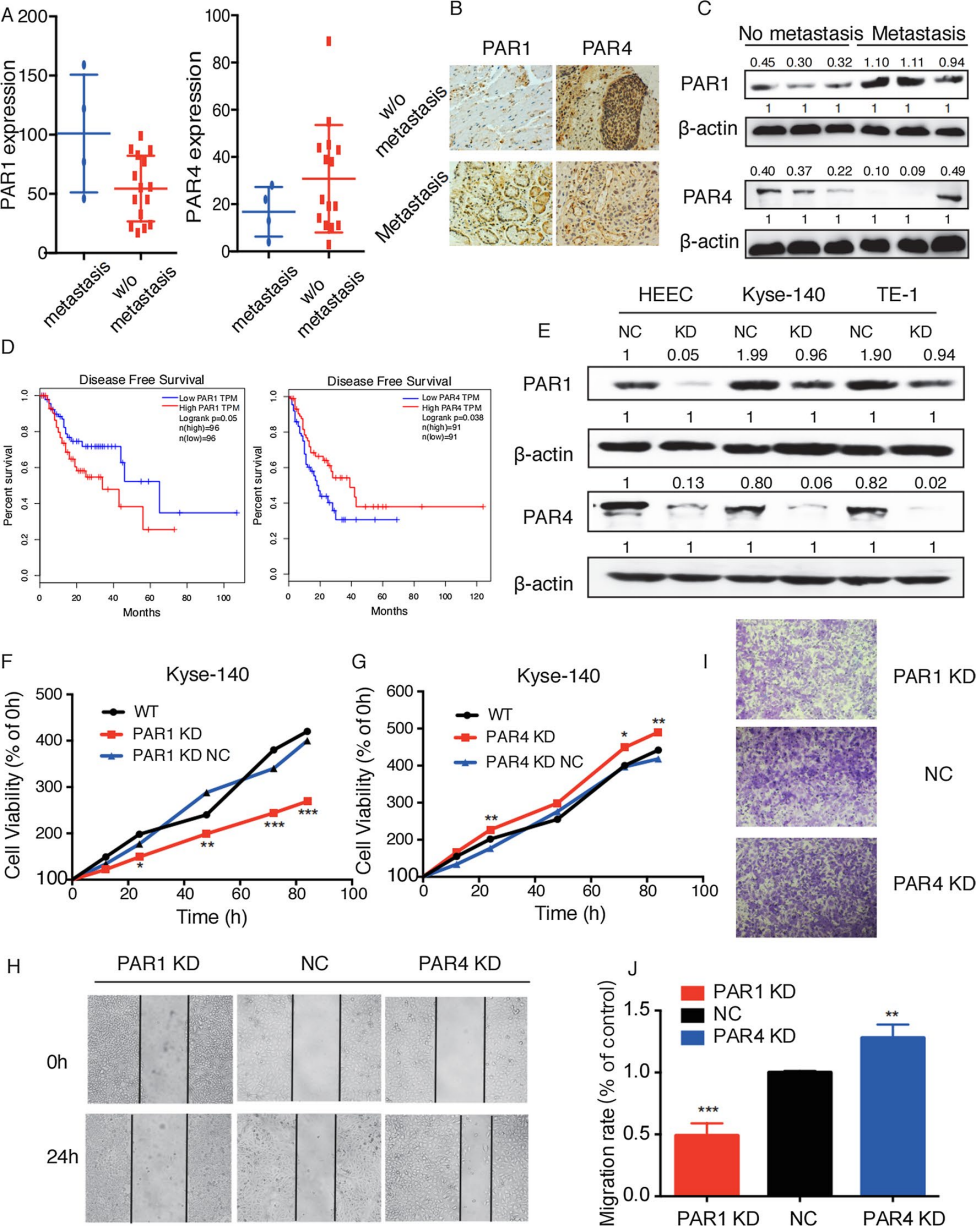
PAR1 exerts an oncogene, while PAR4 exerts a tumor suppressor in ESCC. ** A** IHC detected the expression of PAR1 and PAR4 in ESCC tissues with or without (w/o) metastasis and representative photomicrographs of immunohistochemical staining were shown in **B**. **C** Western blot detected the expression of PAR1 and PAR4 at protein level. **D** Kaplan-Meier analysis of Disease Free Survival (DFS) related to the vWF expression in ESCC patients according to the data downloaded from TCGA database. The log-rank test was used to calculate the DFS rate for comparison between different groups. **E** Western blot detected the expression of PAR1 and PAR4 in HEEC, Kyse140 and TE-1 cells. **F**, **G** The effect of PAR1 or PAR4 KD on Kyse140 proliferation was analyzed by MTT assay. Cell viability was tested at 24th, 48th, 72th and 96th hour after transfection. **H** Wound healing assay detected migration ability of Kyse140 cells transfected with PAR1 or PAR4 shRNA. **I** Transwell assay detected the migration ability of Kyse140. Cells which passed through the Transwell membranes were dissolved in methanol and quantified by microplate reader. OD value at 405nm wavelength was shown in **J**. Data are presented as mean ± SD from three independent experiments; comparison between two groups, *P < 0.05; **P < 0.01; ***P < 0.001. Scale bar: 200 μm in wound healing assay, 100 μm in Transwell assay

To explore the impact of PAR1/PAR4 on cancer metastasis in ESCC, we investigated clinical ESCC specimens with qPCR and IHC assays. As shown in Fig. 2A and B, compared with non-metastatic patients, the expression of PAR1 in patients with metastasis had increased by 50%, while the expression of PAR4 decreased. In addition, tumor tissues derived from patients in late stage III–IV (TNM) showed lower PAR4 levels and higher PAR1 levels, compared with patients in stage II (Additional file 1: Fig. S1A, B). The similar results were also demonstrated with western Blot (Fig. 2C), indicating that PAR1 and PAR4 may play opposite roles in the development of ESCC, and PAR1 may have great impact on cancer metastasis in ESCC. Then, we investigated the correlation between disease free survival (DFS) and the levels of PAR1 and PAR4. The Kaplan-Meier survival analysis revealed that higher PAR1 expression was associated with poor survival, while higher PAR4 expression was linked with prolonged survival of ESCC patients (Fig. 2D).

To further explore the role of PAR1 and PAR4 in ESCC, we transiently knock down PAR1 and PAR4 in HEEC, Kyse140 and TE-1 cells. Western blot results demonstrated effective silencing of PAR1 and PAR4 proteins (Fig. 2E). The effect of PAR1 and PAR4 knockdown on cell proliferation was detected by MTT assay. As shown in Fig. 2F and G, PAR1 knockdown significantly inhibited the proliferation of Kyse-140, while PAR4 knockdown had the opposite effect (Fig. 2F, G) and the results of TE-1 and HEEC cell lines were shown in Additional file 2: Fig. S2A. Flow cytometry and western blot were applied to detect the expression levels of apoptosis-related molecules. The results showed that PAR1 knockdown significantly inhibited the apoptosis of ESCC cell lines, which is opposite of PAR1 knockdown (Additional file 2: Fig. S2C, D). Meanwhile, by comparing the protein levels of PAR1 and PAR4 in patients’ tumor tissues whose diameters were larger than or less than 1000 square millimeters, we found that the expression of PAR1 was upregulated in larger tumors, while PAR4 was downregulated (Additional file 2: Fig. S2B). Then, the effect of PAR1 and PAR4 on cancer metastasis was detected by wound healing and transwell assays. Results revealed that the migration of PAR1 knockdown cell lines was suppressed while the migration of PAR4 knockdown cell lines was promoted, and the results of Kyse140 cell line were shown in Fig. 2H and I, and the results of TE-1 were shown in Additional file 2: Fig. S2E, F).

**Fig. 3:**
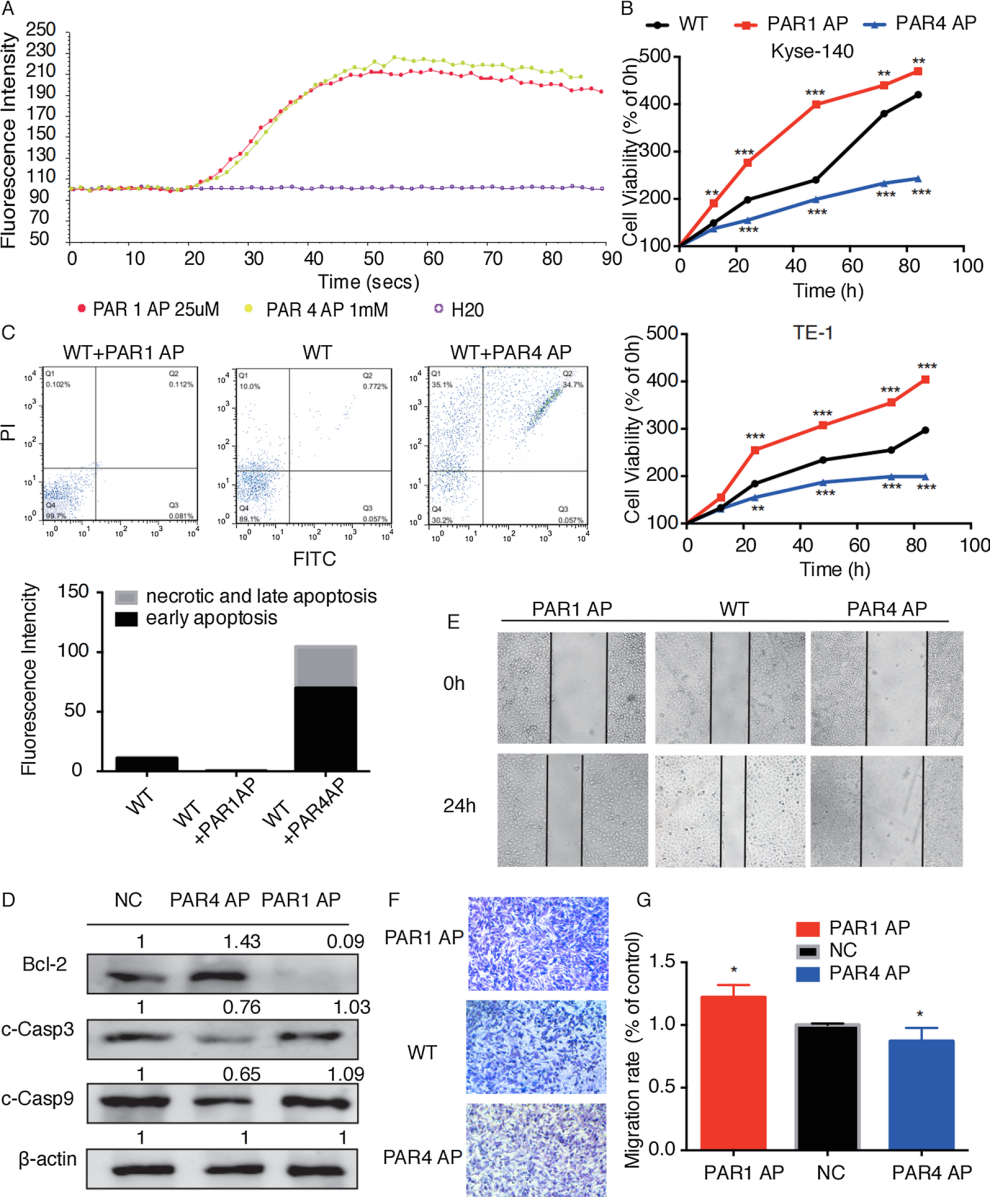
PAR1 promoted the growth and metastasis of ESCC cell lines, while PAR4 had an inhibitory effect in vitro. **A **Fluorescence intensity represented the effects of PAR1/PAR4 on intracellular calcium mobilization in Kyse140 cells. **B** MTT assay detected the effect of PAR1 AP or PAR4 AP on Kyse140 proliferation. Cell viability was tested at 24th, 48th, 72th and 96th h. **C** The FACS detected PAR1 AP or PAR4 AP-induced ESCC cell apoptosis. Percentage of apoptotic cells compared to control was quantitated by mean fluorescence intensity. **D** Western blot analysis of apoptosis-related molecules in Kyse140 cells. β-actin was used as a loading control. **E** Wound healing assay detected migration ability of Kyse140 cells treated with PAR1 AP or PAR4 AP. **F** Transwell assay detected the migration ability of Kyse140. Cells which passed through the Transwell membranes were dissolved in methanol and quantified by microplate reader. OD value at 405nm wavelength was shown in **G**. Data are presented as mean ± SD from three independent experiments; comparison between two groups, *P < 0.05; **P < 0.01; ***P < 0.001. Scale bar: 200 μm in wound healing assay, 100 μm in Transwell assay

Next, we detected the changes of calcium flow, and found that PAR1 and PAR4 AP could activate Kyse140 cell line (Fig. 3A). PAR1 activation significantly enhanced cell growth in both Kyse140 and TE-1 cells, while PAR4 activation had a negative effect on cell growth (Fig. 3B). Then, we revealed that PAR1 AP could slightly inhibit tumor cell apoptosis and PAR4 AP could significantly promote tumor cell apoptosis (Fig. 3C, D). Besides, PAR1 activation also significantly enhanced the migration ability of tumor cells in vitro, while PAR4 activation had an inhibitory effect (Fig. 3E–G). Taken together, these data suggested that PAR1 could potently enhance tumor cell growth and metastasis, while PAR4 had an inhibitory effect in vitro.

#### PAR1 promoted the proliferation and metastasis of ESCC cell lines, while PAR4 had an inhibitory effect in vivo

**Fig. 4:**
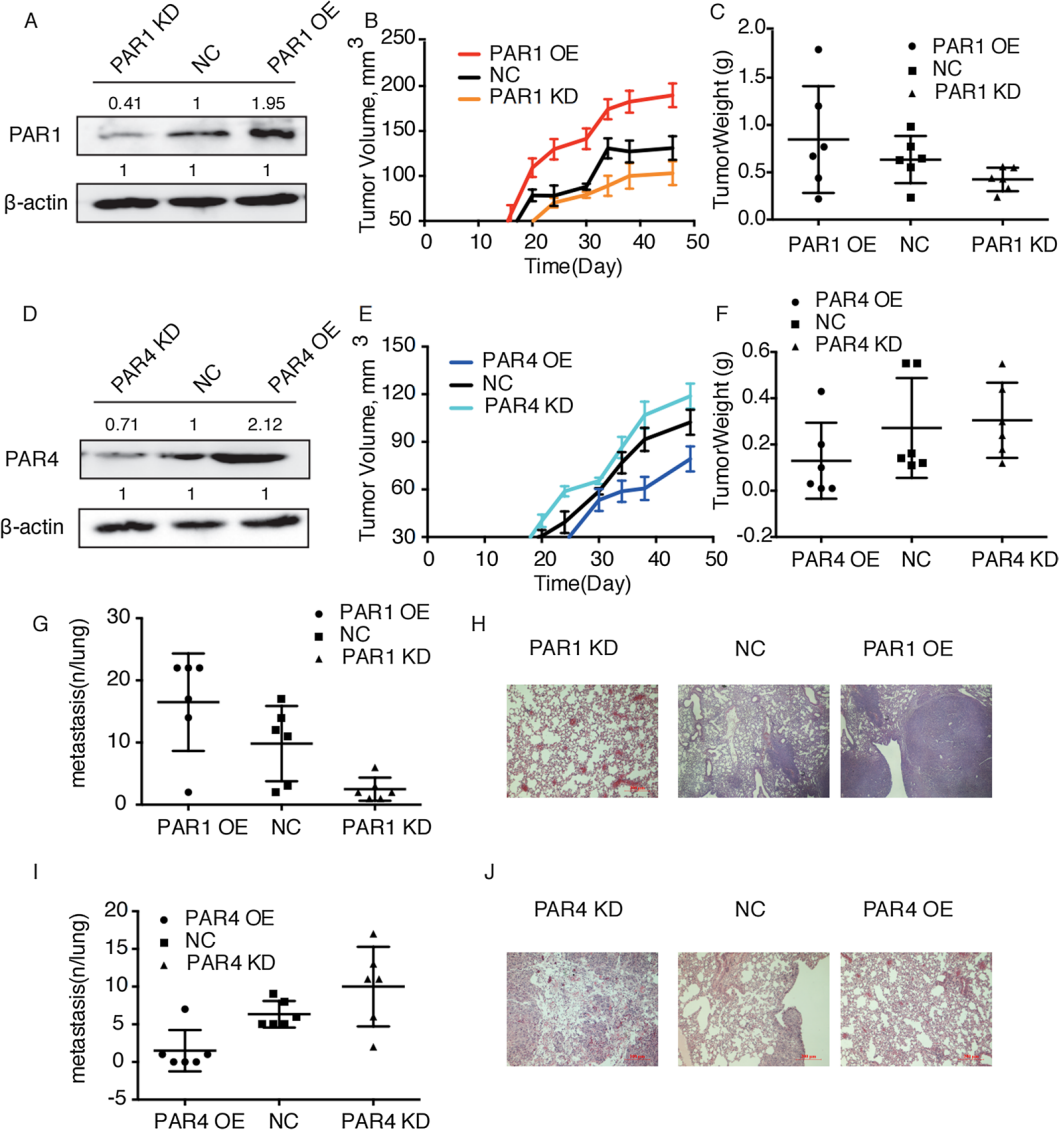
PAR1 promoted the proliferation and metastasis of ESCC cell lines, while PAR4 had an inhibitory effect in vivo. ** A**, **D** Western Blot analysis of PAR1 or PAR4 expression in TE-1 cells after knockdown or overexpression. **B**, **C** PAR1-overexpressed, PAR1-knockdown and control TE-1 cells were injected subcutaneously into mice. Tumor volume was monitored within 50 days (**B**), and tumors were dissected and weighed on the 50th day (**C**). **E**, **F** PAR4-overexpressed, PAR4-knockdown and control TE-1 cells were injected subcutaneously into mice. Tumor volume was monitored within 50 days (**E**), and tumors were dissected and weighed on the 50th day (**F**). **G**, **I** Pulmonary metastasis was observed and recorded 50 days after TE-1 cells injection via tail vein. Average number of lung metastasis in each group was shown. **H**, **J** Representative histologic images of tumor sections from different groups. 4% paraformaldehyde-embedded mice lungs were cut, stained with hematoxylin and eosin, examined histologically and photoed by microcopy. Data are presented as mean ± SD from three independent experiments; comparison between two groups, *P < 0.05; **P < 0.01; ***P < 0.001

**Fig. 5:**
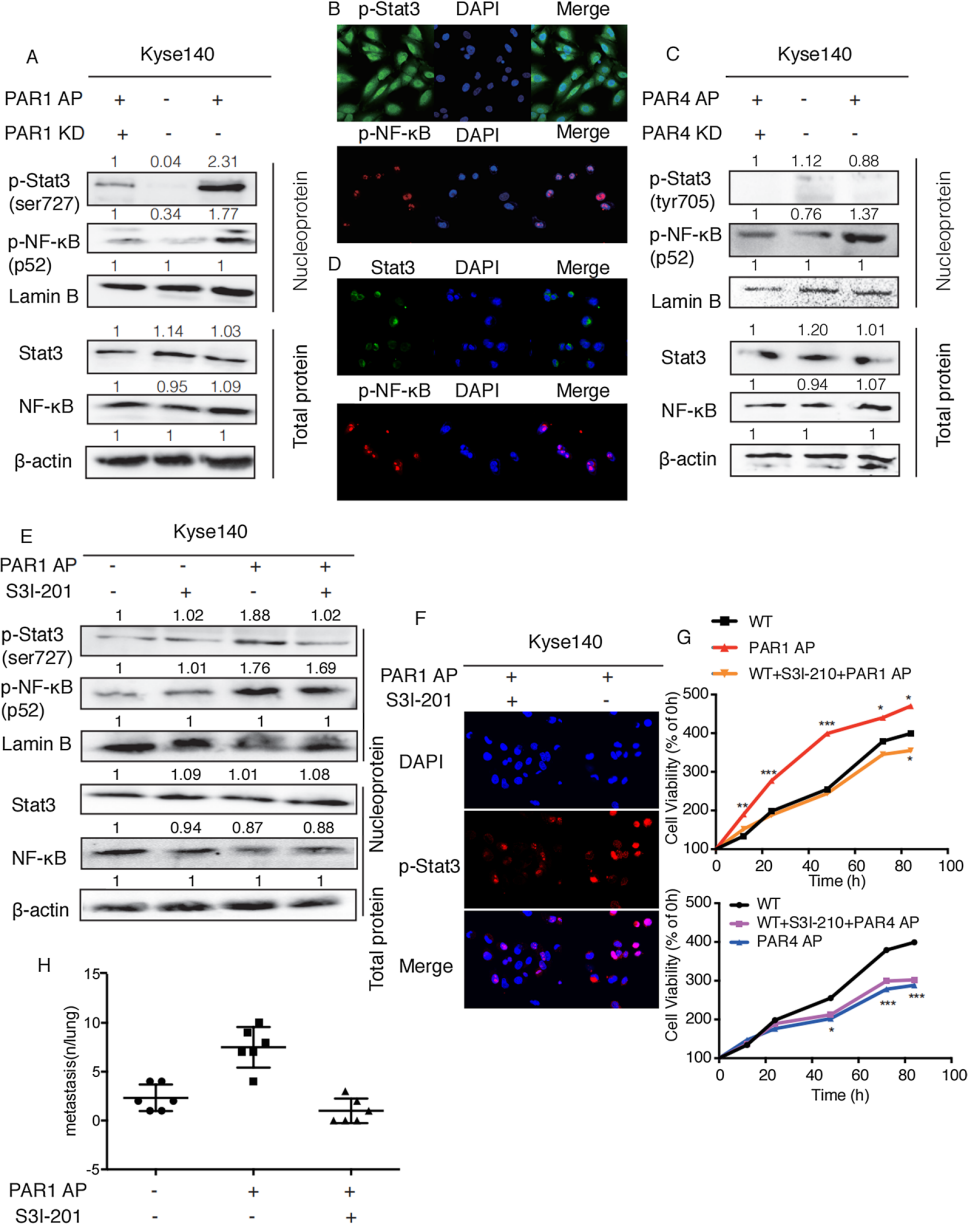
The co-activation of STAT3 and NF-κB is involved in the PAR1 activation-induced tumor promoting effect, while only NF-κB participates in the PAR4 activation-induced tumor inhibitory effect in ESCC. ** A**, **C**, **E** Western blot analyzed the expression of p-STAT3/p-NF-κB in nucleoprotein and STAT3/ NF-κB in total protein of Kyse140 cells. **B**, **D**, **F** Immunofluorescence staining and confocal microscopy detected p-STAT3, STAT3 and p-NF-κB expression in Kyse140 cells. **G** MTT detected the effect of STAT3 inhibitor on Kyse140 proliferation. **H** Pulmonary metastasis was observed and recorded 50 days after Kyse 140 cells injection via tail vein. Average number of lung metastasis in each group was shown. Data are presented as mean ± SD from three independent experiments; comparison between two groups, *P < 0.05; **P < 0.01; ***P < 0.001

In order to further demonstrate the role of PAR1 and PAR4 in tumor metastasis in vivo, we established an experimental metastasis model with female C57BL/6J mice. First, the expression of PAR1 and PAR4 in TE-1 cell line was knocked down by lentivirus infection, and was overexpressed by CRISPR-SAM. The efficiency of knockdown and overexpression was confirmed by western blot (Fig. 4A, D). Consistent with the in vitro observations, as shown in Fig. 4B–F, the PAR1 knockdown cells markedly suppressed the growth of tumor compared with the control mice. The similar results were also found in the mice injected with PAR4 overexpression cells. These findings were also confirmed by counting the number of pulmonary nodules on the lung surface. PAR1 knockdown significantly reduced the number of metastatic nodules, while the PAR1 overexpression markedly increased the number of nodules (Fig. 4G). Besides, PAR4 overexpression significantly inhibited the pulmonary metastasis (Fig. 5I). The similar results were also found with H&E staining, by which we observed the metastatic lesions under a microscope (Fig. 5H, J).

#### The co-activation of STAT3 and NF-κB is involved in the PAR1 activation-induced tumor promoting effect, while only NF-κB participates in the PAR4 activation-induced tumor inhibitory effect

Several studies have illustrated the specificity of NF-κB/STAT3 pathway activation—STAT3 and NF-κB jointly regulate several oncogenes and inflammatory genes [17]. When NF-κB is activated alone, it acts as an tumor suppressor. Recent studies have focused on the relationship between PAR1/PAR4 and NF-κB/STAT3 signaling pathway, which enlightened us that the reason why PAR1 and PAR4 had completely opposite effects on ESCC progress might be attributed to the distinct activation of STAT3 and NF-κB in ESCC cell lines. First, western Blot was applied to investigate the impact of PAR1 AP on STAT3 and NF-κB levels in the cell nucleus. As shown in Fig. 5A, PAR1 activation by AP obviously increased the protein levels of p-STAT3 and p-NF-κB in the nucleus, which was reversed by PAR1 knockdown. We also obtained such discovery under a confocal microscopy (Fig. 5B). Then, We detected the impact of PAR4 AP on STAT3 and NF-κB levels in the nucleus. As shown in Fig. 5C, the activation or knockdown of PAR4 had no effect on the expression levels of p-STAT3 in the nucleus, but the activation of PAR4 significantly enhanced the expression levels of p-NF-κB in the nucleus, which was reversed by PAR4 knockdown. Similarly, we also found that in PAR4 AP-activated ESCC cells, STAT3 was mostly distributed in the cytoplasm, while p-NF-κB was in the nucleus (Fig. 5D). Then, we applied STAT3 inhibitor S3I-201 to explore whether STAT3 activation and the nuclear translocation of p-STAT3 have effects on the PAR1 activating-induced cancer promoting effect. Western blot and immunofluorescence results showed that S3I-201 could inhibit the expression of p-STAT3 in the nucleus induced by PAR1 AP (Fig. 5E, F). Meanwhile, S3I-201 significantly reversed the promoting effect of PAR1 AP on the proliferation of ESCC cells, but had no effect on the suppressive proliferation of ESCC cells induced by PAR4 AP (Fig. 5G). In addition, the results of in vivo metastasis model suggested that compared with the mice injected with PAR1 AP alone, the number of pulmonary metastatic nodules in PAR1AP and S3I-201 co-injection group was significantly reduced (Fig. 5H).

### nSMase2/MAPK/NF-κB mediated PAR4 activation-induced tumor inhibitory effect

**Fig. 6:**
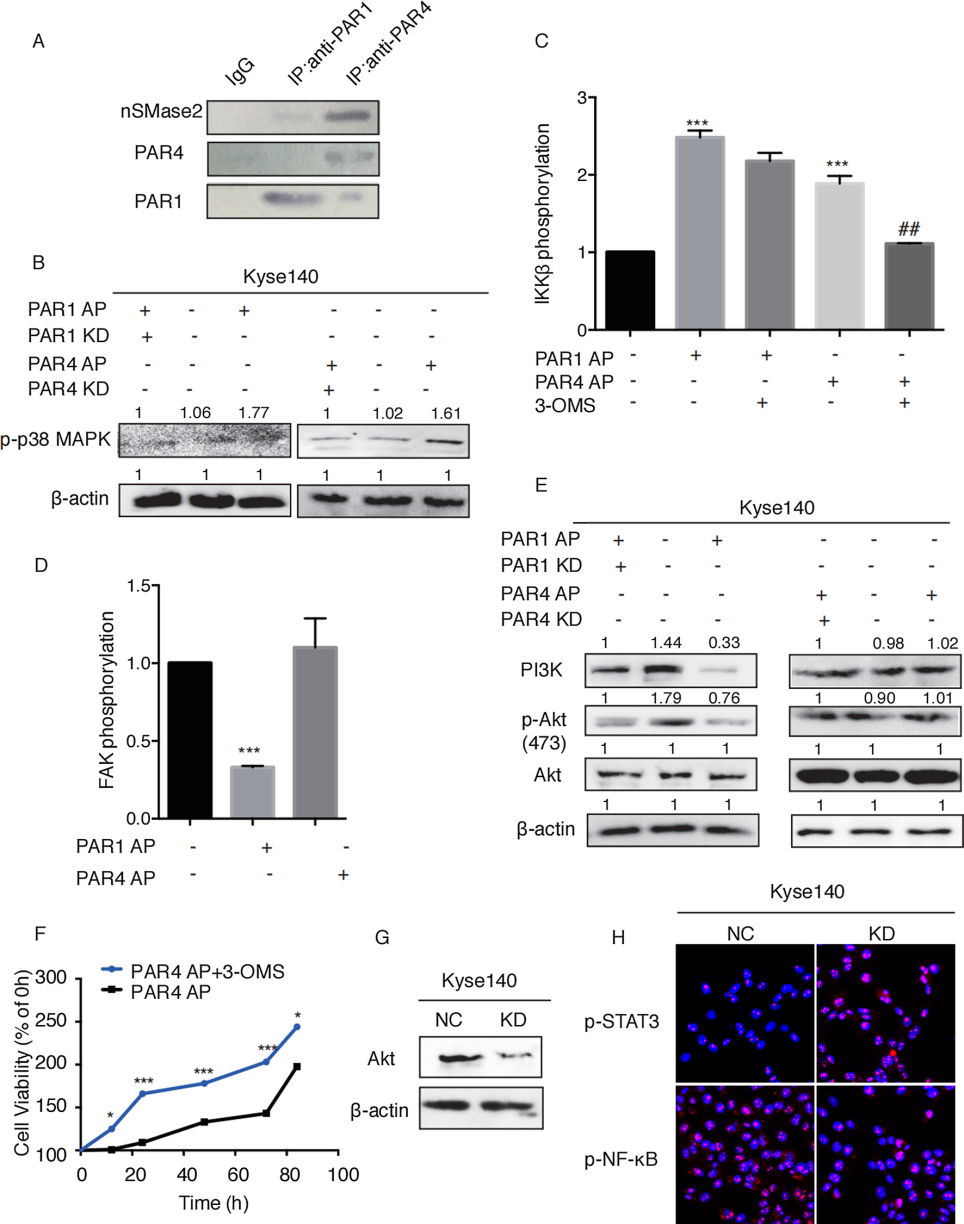
nSMase2/MAPK/NF-κB mediated PAR4 activation-induced tumor inhibitory effect, and FAK/PI3K/AKT/STAT3/NF-κB mediated PAR1 activation-induced tumor promoting effect in ESCC. **A **Kyse140 cells were lysed and immunoprecipitated with antibodies against IgG control, PAR1 or PAR4 and then immunoblotted with antibodies against nSMase2, PAR1 or PAR4. **B** Western blot detected the expression of p-p38 MAPK. **C** Kyse140 cells were pre-incubated with 30µM 3-OMS, followed by the addition of either PAR1-AP or PAR4 AP to trigger the phosphorylation of IKKβ. The IKKβ phosphorylation was detected by FCM. **D** Kyse140 cells were pre-treated with PAR1 AP or PAR4 AP. FAK phosphorylation was detected by FCM. **E** Western blot detected the expression of PI3K, p-Akt and Akt. **F** MTT detected the effect of nSMase inhibitor on Kyse140 proliferation. **G** Western blot verified the knockdown efficiency of shAKT plasmids. **H** Immunofluorescence staining and confocal microscopy detected p-STAT3 and p-NF-κB expression in control and AKT-knockdown Kyse140 cells. Data are presented as mean ± SD from three independent experiments; comparison between two groups, *P < 0.05; **P < 0.01; ***P < 0.001

Based on the preliminary experiments and literature, we put forward a hypothesis that nSMase2 might play a role in the activation of NF-κB induced by PAR4 AP. Kyse140 cells were immunoprecipitated with anti-PAR1 or anti-PAR4 antibodies, followed by immunoblotting with anti-nSMase2 antibody. As shown in Fig. 6A, nSMase was directly associated with PAR4 but not PAR1. We also speculated whether the different effects of PAR1 and PAR4 on ESCC were related to the activation of different signaling pathways. Then, western blot detected the expression levels of p-p38 MAPK under the conditions of PAR1/PAR4 activation or silence. The results showed that the expression of p-p38 MAPK was upregulated by both PAR1 and PAR4 activation (Fig. 6B). Then, flow cytometry was applied to demonstrate that the levels of p-IKKβ were increased by PAR1 or PAR4 activation. However, we found that 3-OMS (a nSMase inhibitor) almost completely abolished IKKβ phosphorylation induced by PAR4 AP but did not affect phosphorylation induced by PAR1-AP (Fig. 6C).

Next, we explored the FAK phosphorylation following PAR1 AP or PAR4 AP treatment. As shown in Fig. 6D, PAR1 activation, but not PAR4 activation, downregulated the levels of p-FAK. Meanwhile, PAR1 activation had a significant inhibitory effect on the expression of PI3K and p-Akt, while PAR4 exerted little effect (Fig. 6E). MTT assays showed that nSMase2 activation was involved in PAR4-mediated tumor inhibitory effect and nSMase2 inhibitor 3-OMS weakened this effect induced by PAR4 AP (Fig. 6F). To sum up, PAR4 activation induced nSMase2/MAPK pathway, and IKKβ phosphorylation promoted NF-κB phosphorylation and nuclear translocation, and then produced the anti-cancer effect.

#### FAK/PI3K/AKT/STAT3/NF-κB mediated PAR1 activation-induced tumor promoting effect

In order to further explore whether AKT played a role in PAR1-induced tumor promoting effect, we first constructed AKT knockdown Kyse140 cell line (Fig. 6G). Immunofluorescence results indicated that PAR1 AP induced the distribution of p-STAT3 in the nucleus, while the distribution of p-NF-κB in the nucleus was slightly affected (Fig. 6H). To sum up, PAR1 activation inhibited FAK phosphorylation and further suppressed PI3K/AKT, thereby inducing the nucleus translocation of p-STAT3, and then exerted an tumor promoting effect.

## Discussion

PARs have been demonstrated to regulate tumor growth, invasion and metastasis in a variety of malignant tumors [18–20]. PAR1, which is widely expressed in human cancers, promotes the transformation and adhesion of pancreatic cancer cells and the invasion and metastasis of oral adenocarcinoma, colon cancer and breast cancer cells [21–27]. PAR2 is closely related to the growth and invasion of nasopharyngeal cancer, breast cancer, gastric cancer, colon cancer, prostate cancer and pancreatic cancer [28–35]. PAR1 and PAR2 are upregulated in ESCC, and can be served as possible prognostic markers because their expression levels are closely linked with the stage of tumor [36]. The latest study has shown that compared with paired non-cancerous tissues, the expression of PAR4 in gastric cancer tissues is significantly reduced, especially in tumors with lymph node or poorly differentiated tumors. Jiang et al. [37]. have reported that the down-regulated expression of PAR4 was found in lung adenocarcinoma, and the decrease of PAR4 levels was associated with a more aggressive clinical phenotype, suggesting that PAR4 may act as a tumor suppressor in lung adenocarcinoma. However, PAR4 is overexpressed in colorectal cancer [38] and liver cancer [39], and the overexpression of PAR4 contributes to the proliferation of cancer cells [38]. In addition, PAR4 agonists can induce calcium influx and promote the proliferation of colon cancer cells through ErbB2 transcription activation and Src kinase pathway [40]. It has been reported PAR4 is frequently down-regulated in ESCC specimens which is partly due to the hypermethylation of the PAR4 promoter [15]. In our study, we collected and detected clinical samples and found that PAR4 levels were decreased and PAR1 levels were increased in esophageal squamous cancer, compared with paired non-cancerous tissues. Besides, the levels of PAR4 and PAR1 were related to tumor size and distant metastasis. Then, we noted that PAR4 exerts an tumor suppressor and PAR1 has the opposite effect in ESCC which is confirmed by both in vitro and in vivo experiments. Therefore, PAR1 and PAR4 might be potential biomarkers for ESCCdiagnosis and prognosis. Wang et al. [41] employed a TMT-based quantitative proteomic approach to identify PAR4-regulated proteomic profiles in ESCC cells and showed that key higher expressed proteins included those linked with apoptosis and tumor suppressors (e.g. caspase 9), and lower expressed proteins included those linked with anti-apoptosis, autophagy and enhancing cell proliferation. Their results of bioinformatics analysis are consistent with our *in vitro* and *in vivo* findings. Wang’s group also suggested PAR4 activation (PAR4-AP) could inhibit the viability and induce the apoptosis of ESCC cells through suppressing enrichments of DNMT1 and HDAC2 at the p16 promoter via MAPK signals [42].

Moreover, we explored the role of STAT3/NF-κB pathway in the PAR1/PAR4 activation-induced tumor promoting/inhibitory effect. The experimental results revealed that PAR1 activation induced the nuclear translocation of STAT3 and NF-κB while PAR4 activation could only induce the nuclear translocation of NF-κB. Then, we further confirmed the role of the FAK/PI3K/AKT pathway in PAR1 activation-induced nucleus translocation of STAT3 and the role of nSMase2/MAPK pathway in PAR4 activation-induced nucleus translocation of NF-κB. In addition, it is well known that PAR1 and PAR4 are calcium channel proteins. We found that PAR1 and PAR4 activation induced significant changes in calcium flow of ESCC cells. Therefore, the intracellular pathways that mediate PAR1 and PAR4 activation-induced changes in calcium flow are worth further investigating in our future research (Additional file 3).

## Conclusions

**Fig. 7:**
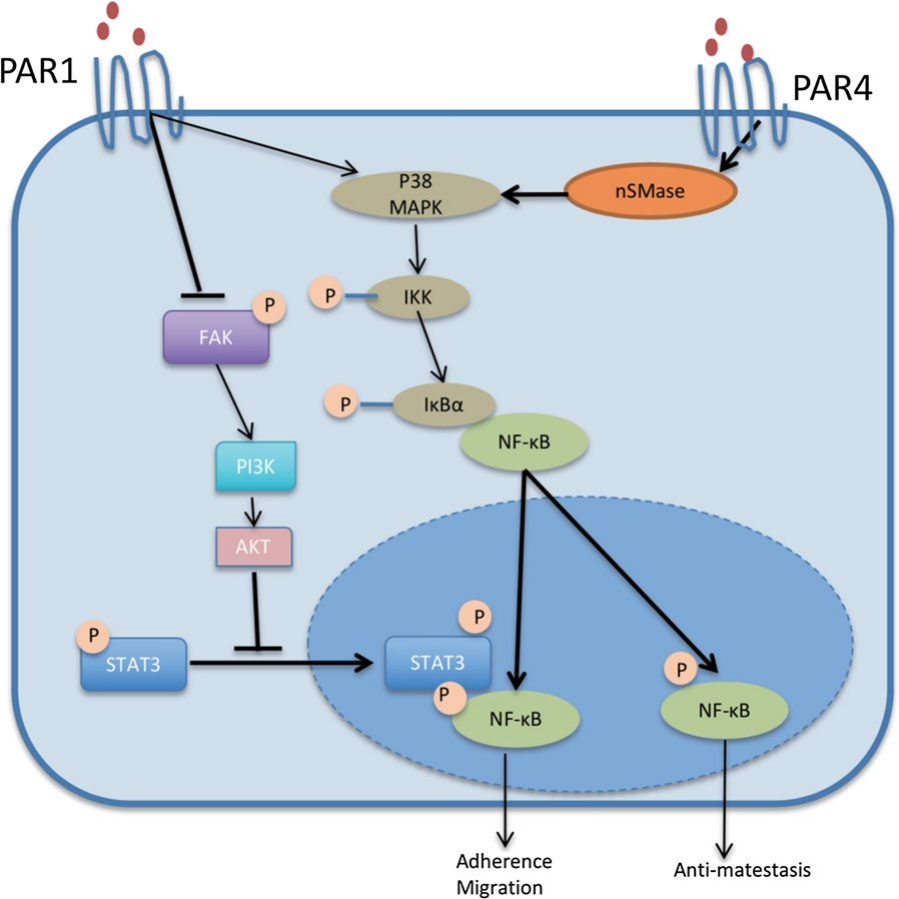
The schematic illustration of the mechanism of PAR1- and PAR4-induced tumor promoting/inhibitory effects in ESCC

In brief, as shown in Fig. 7, nSMase2/MAPK/NF-κB pathway mediated PAR4 activation-induced tumor inhibitory effect, while FAK/PI3K/AKT/STAT3/NF-κB pathway mediated PAR1 activation-induced tumor promoting effect in ESCC.

## Supplementary Information

**Additional file 1: Fig. S1.** Immunohistochemistry detected PAR1 or PAR4 expression in ESCC tissues (stage II/III/IV) and representative photomicrographs of IHC staining was shown in (B).

**Additional file 2: Fig. S2.** (A) MTT assay detected the effect of PAR1 or PAR4 knockdown (KD) on TE-1 and HEEC cell proliferation. Cell viability was tested at 24th, 48th, 72th and 96th hour. (B) Immunohistochemistry detected PAR1 or PAR4 expression in ESCC tissues (tumor volume above or below 1000mm^3^). (C) Western blot analysis of apoptosis-related molecules in TE-1 cells. β-actin was used as a loading control. (D) The FACS detected PAR1 or PAR4 KD-induced ESCC cell apoptosis. Percentage of apoptotic cells compared to control was quantitated by mean fluorescence intensity. (E) Wound healing assay tested the migration ability of TE-1 cells transfected with PAR1 or PAR4 siRNA. (F) Transwell assay detected the migration ability of TE-1 cells. Data are presented as mean ± SD from three independent experiments; comparison between two groups, *P < 0.05; **P < 0.01; ***P < 0.001. Scale bar: 200 μm in wound healing assay, 100 μm in Transwell assay.

**Additional file 3: Fig. S3.** A-I. The semi-quantitative analysis of western blotting via Image J software.

## Data Availability

The data that support the findings of this study are available from the corresponding author upon reasonable request.
