# Supplementary figures and images for: PAR1 and PAR4 exert opposite effects on tumor growth and metastasis of esophageal squamous cell carcinoma via STAT3 and NF-κB signaling pathways
Source: Cancer Cell Int. 2021 Nov 29;21:637. doi: 10.1186/s12935-021-02354-4 (PMC8628382; doi:10.1186/s12935-021-02354-4)

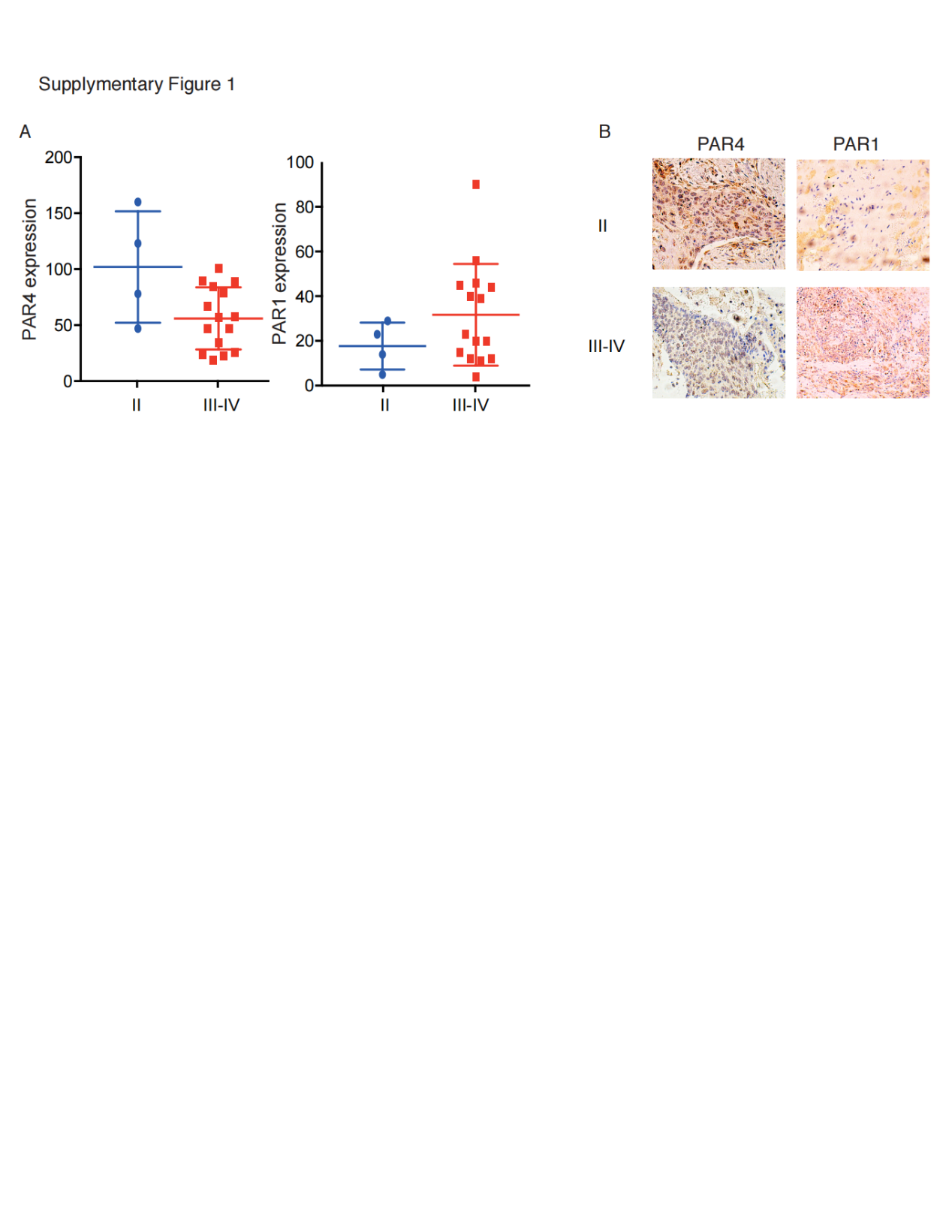

Supplement: Supplementary file 1 — Additional file 1: Fig. S1. Immunohistochemistry detected PAR1 or PAR4 expression in ESCC tissues (stage II/III/IV) and representative photomicrographs of IHC staining was shown in (B). [file 12935_2021_2354_MOESM1_ESM.docx]

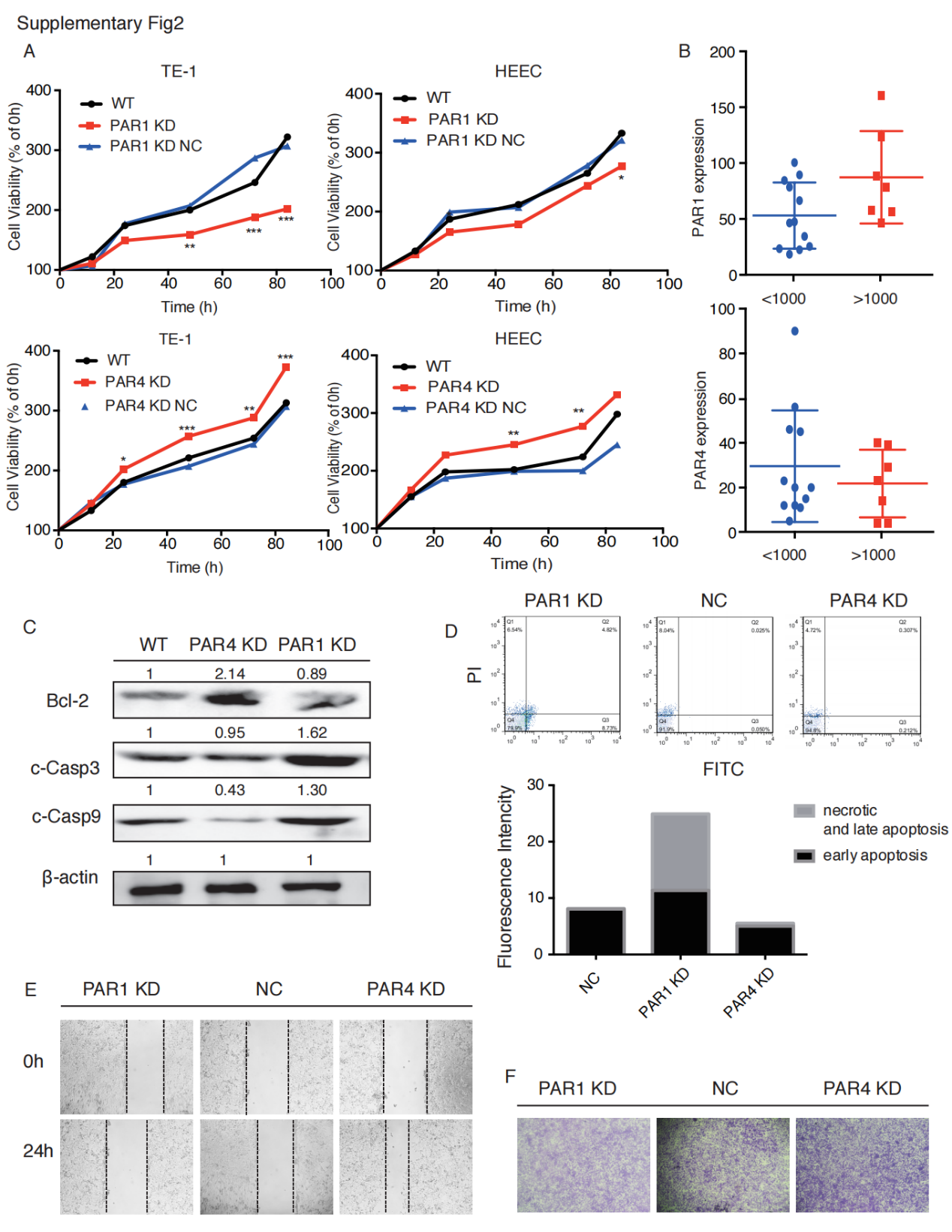

Supplement: Supplementary file 2 — Additional file 2: Fig. S2. (A) MTT assay detected the effect of PAR1 or PAR4 knockdown (KD) on TE-1 and HEEC cell proliferation. Cell viability was tested at 24th, 48th, 72th and 96th hour. (B) Immunohistochemistry detected PAR1 or PAR4 expression in ESCC tissues (tumor volume above or below 1000mm3). (C) Western blot analysis of apoptosis-related molecules in TE-1 cells. β-actin was used as a loading control. (D) The FACS detected PAR1 or PAR4 KD-induced ESCC cell apoptosis. Percentage of apoptotic cells compared to control was quantitated by mean fluorescence intensity. (E) Wound healing assay tested the migration ability of TE-1 cells transfected with PAR1 or PAR4 siRNA. (F) Transwell assay detected the migration ability of TE-1 cells. Data are presented as mean ± SD from three independent experiments; comparison between two groups, *P < 0.05; **P < 0.01; ***P < 0.001. Scale bar: 200 μm in wound healing assay, 100 μm in Transwell assay. [file 12935_2021_2354_MOESM2_ESM.docx]

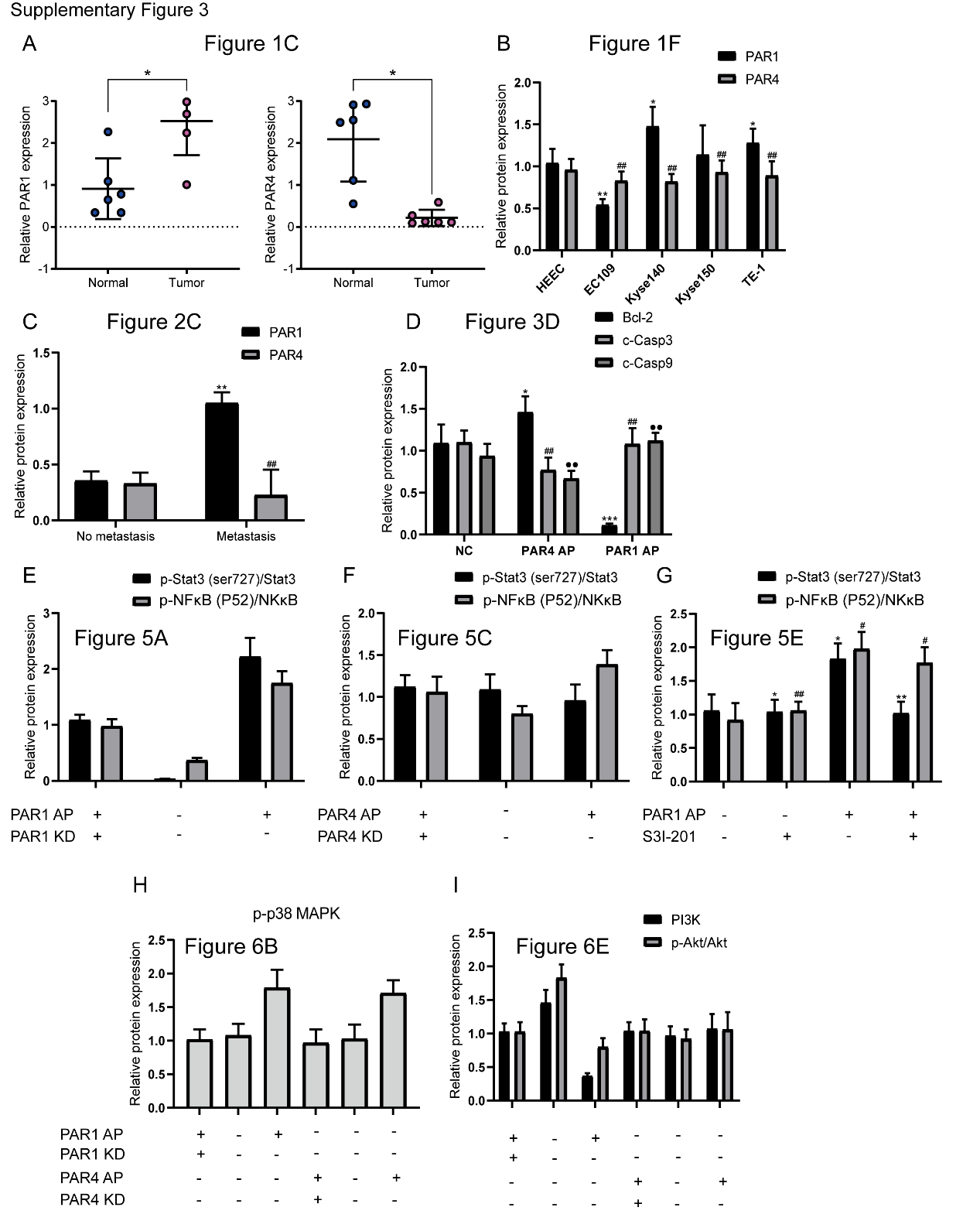


Figure S3

Supplement: Supplementary file 3 — Additional file 3: Fig. S3. A-I. The semi-quantitative analysis of western blotting via Image J software. [file 12935_2021_2354_MOESM3_ESM.docx]
